# Aptamer Cocktail to Detect Multiple Species of Mycoplasma in Cell Culture

**DOI:** 10.3390/ijms21113784

**Published:** 2020-05-27

**Authors:** Quanyuan Wan, Xiaohui Liu, Zihua Zeng, Zhenghu Chen, Yanting Liu, Youli Zu

**Affiliations:** Department of Pathology and Genomic Medicine, Houston Methodist Hospital, 6565 Fannin Street, Houston, TX 77030, USA; qwan@houstonmethodist.org (Q.W.); xliu2@houstonmethodist.org (X.L.); zzeng@houstonmethodist.org (Z.Z.); zchen2@houstonmethodist.org (Z.C.); yanting.liu@foxmail.com (Y.L.)

**Keywords:** DNA aptamer, mycoplasma, cell culture, rapid detection

## Abstract

Mycoplasma contamination of cell line cultures is a common, yet often undetected problem in research laboratories. Many of the existing techniques to detect mycoplasma contamination of cultured cells are time-consuming, expensive, and have significant drawbacks. Here, we describe a mycoplasma detection system that is useful for detecting multiple species of mycoplasma in infected cell lines. The system contains three dye-labeled detection aptamers that can specifically bind to mycoplasma-infected cells and a dye-labeled control aptamer that minimally binds to cells. With this system, mycoplasma-contaminated cells can be detected within 30 min by using a flow cytometer, fluorescence microscope, or microplate reader. Further, this system may be used to detect mycoplasma-contaminated culture medium. This study presents an novel mycoplasma detection model that is simple, rapid, inexpensive, and sensitive.

## 1. Introduction

Mycoplasma cell line contamination is an intractable and serious problem in biological experiments. Due to their size, mycoplasma cannot be visualized and therefore, can affect the results of cell experiments in imperceptible ways, leading to erroneous or misleading results [1]. Currently, the scientific community is considering whether mycoplasma testing should be a mandatory element in any cell culture laboratory [2,3,4,5]. To prevent mycoplasma contamination, all new cell lines entering the lab should be screened, and all cells in culture should be monitored routinely, especially before initiating a long-term experiment or cell cryopreservation. As the importance of mycoplasma surveillance has become more widely acknowledged, numerous methods, protocols, and even commercial kits have been developed to detect mycoplasma [6,7,8,9]. Most are based on a polymerase chain reaction (PCR) assay, which is characterized as sensitive and specific, but also troublesome and time-consuming. Therefore, we sought to develop rapid and easy mycoplasma detection methods to benefit laboratory and clinical research endeavors.

Nucleic acid aptamers are single-stranded DNA (ssDNA) or RNA molecules that fold into sequence-specific 3D structures that strongly and specifically bind to target molecules [10,11]. These binding properties make aptamers “chemical antibodies” with several advantages over traditional antibodies. For example, aptamers can be produced using Systemic Evolution of Ligands by Exponential Enrichment (SELEX), which is faster and more economical than the screening procedures for antibody production [12]. Further, SELEX ensures aptamers have high affinities for their targets with Kd values in the low picomolar to micromolar range, allowing them to be used as effective detection probes with minimal dosing [11]. More importantly, with or without a specific target molecule, aptamers can be generated for a wide variety of targets including organic and inorganic molecules, viruses, bacteria, parasites, live or dead cells, and even tissues [11,13,14]. Due to their unique properties, aptamers have broad potential applications in cellular biology, medical diagnostics, and gene therapy [14,15].

Recently, we identified an ssDNA aptamer probe, A15-1, that specifically binds and rapidly detects *Mycoplasma hyorhinis*-infected cells [16]. To further develop potential biomedical applications of this aptamer, we used flow cytometry assays and an aptamer cocktail to detect a wide range of mycoplasma-infected cell samples. In addition, we investigated whether an aptamer cocktail could be used to detect free mycoplasma products in culture media.

## 2. Results

### 2.1. Refining A15-1 Aptamer Binding Conditions for Flow Cytometry Analyses

Our previous study showed that the A15-1 aptamer can bind to *M. hyorhinis*-infected cells, allowing rapid detection. To reduce testing costs and simplify the protocol, we optimized the reaction conditions. We first tested the effect of A15-1 aptamer concentration and reaction time on its cell-binding ability using *M. hyorhinis*-infected KM-H2, HDLM2, Jeko-1, Jurkat, Mino, and Raji cells. We confirmed cell infection status using a standard PCR assay (Figure S1A). The gating strategy used for flow cytometry is shown in Figure S1B. The results showed that A15-1 cell-binding ability did not change significantly with varying A15-1 aptamer concentration (50–400 nM) (Figure S1C). Maximal cell binding by A15-1 aptamer probes occurred in 10 min and was consistent through reaction times lasting up to 60 min (Figure S1D). These findings indicated that the optimal cell-binding conditions for A15-1 require a 50 nM final concentration and 10 min duration reaction time.

Next, we assessed whether bovine serum albumin (BSA) or tRNA were necessary additions to the binding buffer or whether the binding reaction could occur in PBS buffer alone. As seen in Figure S1E,F, tRNA weakened A15-1 binding with *M. hyorhinis* positive (MP^+(hyo)^) Mino and Jeko-1 cells but not Jurkat cells, while BSA did not affect A15-1 binding ability. Additionally, as seen in Figure S1G,H, the binding ability of A15-1 in PBS was similar in both PBS and RPMI-1640 medium, though Mg^2+^ impaired the binding ability slightly. These results suggest that a feasible binding buffer for the A15-1 aptamer is 1 × PBS without Mg^2+^, Ca^2+^, tRNA, or BSA.

### 2.2. Detection of M. hyorhinis–Infected Cells by Flow Cytometry

**Figure 1:**
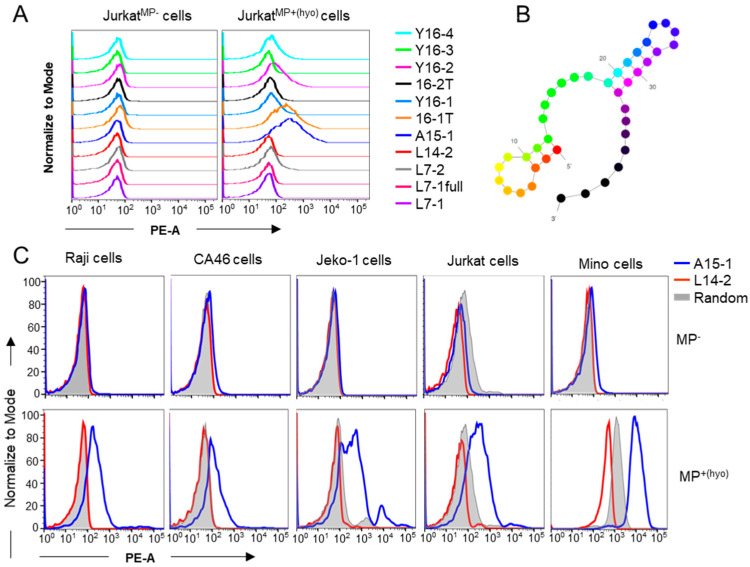
Detection of *M. hyorhinis* infection by flow cytometry assay. PBS-washed cells were incubated with 50 nM of various Cy3-labeled aptamers in 1× PBS for 10 min and binding ability was analyzed using flow cytometry. (**A**) Binding tests of the different aptamers with MP^−^ and MP^+(hyo)^ Jurkat cells; (**B**) predicted secondary structure of L14-2 aptamer; and (**C**) binding tests of A15-1, L14-2, and random library oligos with various MP^−^ and MP^+^ cells. MP^−^, mycoplasma negative; MP^+(hyo)^, *M. hyorhinis* positive. Data show the representative results from three independent experiments.

We have previously shown that the A15-1 aptamer selectively binds *M. hyorhinis*-infected cells [16]. To rule out pseudo-positive results due to non-specific binding of ssDNA sequences to cells [17], we selected an ssDNA sequence for a background signal control. To this end, we screened previously synthesized Cy3-labeled ssDNA oligos available in our library. We found that the L14-2 aptamer did not bind to either mycoplasma negative (MP^−^) or MP^+(hyo)^ Jurkat cells (Table S1 and Figure 1A). The secondary structure of L14-2 is shown in Figure 1B. To confirm that L14-2 was an ideal control aptamer, we compared the binding abilities of A15-1, L14-2, and 76-base random library ssDNA oligos to MP^−^ and MP^+(hyo)^ Raji, CA46, Jeko-1, Jurkat, and Mino cells that were being cultured in our laboratory. As seen in Figure 1C, L14-2 had similar binding ability to MP^−^ cells as A15-1 and the random library, but a lower binding ability than A15-1 and the random library oligos to MP^+(hyo)^ cells, suggesting that L14-2 is a suitable control aptamer that works with A15-1 to detect mycoplasma contamination in cell samples.

### 2.3. Detection of M. hyorhinis Contamination Using a Microplate Reader

Our flow cytometry results demonstrated that the fluorescence intensity of A15-1-bound MP^+(hyo)^ cells was much higher than that of control L14-2-bound MP^+^ cells, while the fluorescence intensities were comparable between A15-1-bound and L14-2-bound MP^−^ cells. To further simplify detection steps, we measured the fluorescence intensity of cell samples pre-incubated with Cy3-labeled aptamers using a microplate reader. Here, we took Jeko-1 and Jurkat cells as the primarily used cells, due to mycoplasma-contaminated Jeko-1 and Jurkat cells were sensitive and less sensitive to be detected by the testing aptamers, respectively. Unexpectedly, the ratios of the relative fluorescence units (RFU) between A15-1-bound and L14-2-bound MP^+/−^ cells were not constant but increased with cell number (Figure S2). As such, we determined that it was impractical to define a cut-off value for the demarcation of MP^+^ or MP^−^ cells. We attempted to optimize testing conditions, including binding and washing, but observed no substantial improvement in the RFU ratio (data not shown). We concluded that the L14-2 aptamer was an unsuitable control aptamer for the microplate reader detection method.

**Figure 2:**
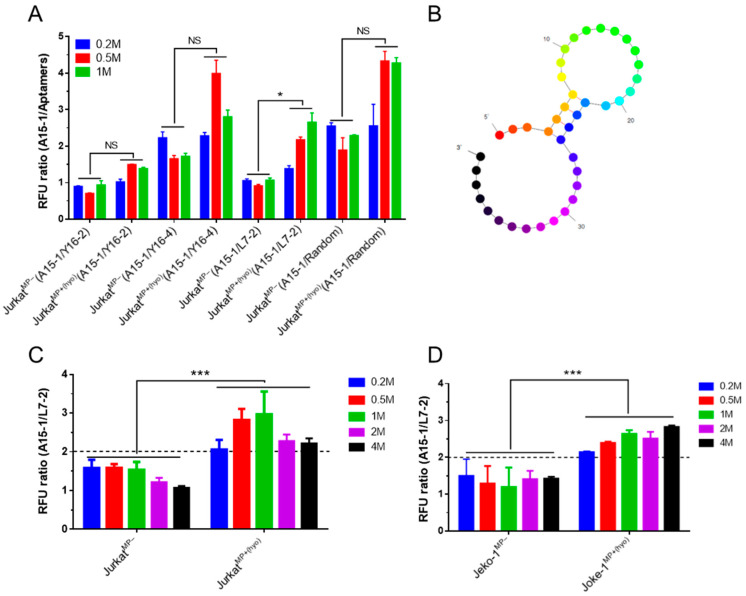
Detection of *M. hyorhinis*-infected cells using a microplate reader. Different counts (0.2, 0.5, 1, 2, or 4 million) of PBS-washed cells were incubated with (50 nM) corresponding aptamers in 1× PBS for 10 min, followed by washing with 1 × PBS containing 0.01% Tween-20 and fluorescence intensity measurement using a microplate reader. (**A**) Screen for the control aptamer using different counts (0.2, 0.5, or 1 million) of Jurkat cells. The RFU ratios between A15-1- and different control aptamer-bound MP^−^ or MP^+(hyo)^ cells were calculated; (**B**) predicted secondary structure of the L7-2 aptamer; (**C**,**D**) MP^−^ and MP^+(hyo)^ Jeko-1 (**C**) and Jurkat (**D**) cells can be distinguished by the RFU ratio between A15-1- and L7-2-bound cells. The horizontal dashed line is the cut-off value line that demarcates MP^−^ and MP^+(hyo)^ cells. MP^−^, mycoplasma negative; MP^+(hyo)^, *M. hyorhinis* positive. Data show the mean ± SD of three independent experiments. * *p* < 0.05, *** *p* < 0.001, “NS” means not significant.

We next screened for an alternative control aptamer. We screened Y16-2, Y16-4, L7-2, and the random library as control aptamer candidates (Figure 1A). As before, we measured the fluorescence intensity of various aptamer-bound cells using a microplate reader and calculated RFU ratios. The RFU ratios between A15-1-bound and L7-2-bound cells were constant and lower in MP^−^ Jurkat cells than in MP^+^ Jurkat cells (Table S1 and Figure 2A). We next predicted the secondary structure of the L7-2 aptamer (Figure 2B) and used Jurkat and Jeko-1 cells to test whether the L7-2 aptamer could serve as an appropriate control aptamer for mycoplasma detection using a microplate reader. The RFU ratios between A15-1-bound and L14-2-bound MP^−^ cells were below 2, while the ratios were above 2 for MP^+^ cells (Figure 2C). These results allowed us to determine a cut-off value to demarcate MP^−^ and MP^+^ cells and established the L7-2 aptamer as a suitable control for our microplate reader detection method.

### 2.4. A15-1 Detects M. hyorhinis but Not Other Mycoplasma Infections

**Figure 3:**
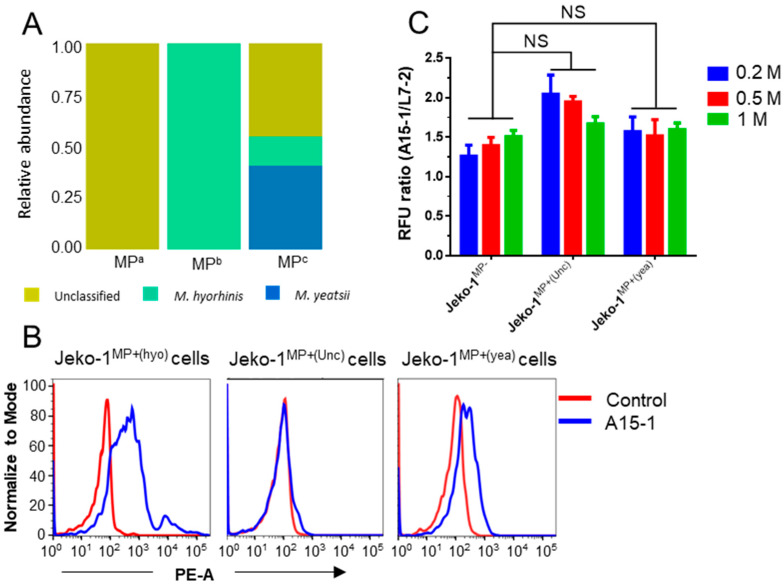
A15-1 aptamer detects *M. hyorhinis* but not other mycoplasma infections. (**A**) Relative abundance of mycoplasma species from the contaminated cells of other laboratories. MP^a^, MP^b^, and MP^c^ represent different species of mycoplasma from the three laboratories, respectively; (**B**) A15-1 (blue) binding test in other species of mycoplasma-infected Jeko-1 cells by flow cytometry. The control aptamer is L14-2 (red); (**C**) Fluorescence intensity measurement of A15-1 or L7-2 (control) aptamer-bound MP^−^ or MP^+^ Jeko-1 cells using a microplate reader. MP^−^, mycoplasma negative; MP^+(hyo)^, *M. hyorhinis* positive; MP^+(Unc)^, unclassified mycoplasma negative; MP^+(yea)^, mixed mycoplasma positive. Data show the mean ± SD of three independent experiments. “NS” means not significant.

We next investigated whether A15-1/L14-2 and A15-1/L7-2 could be used to detect cell contamination from other species of mycoplasma. We collected three mycoplasma-contaminated cell lines from other laboratories and subsequently verified them using PCR detection assays (Figure S3A). To identify the mycoplasma species of contaminated cells, we isolated and prepared whole genomic DNA from the cell samples for 16S rRNA sequencing (Figure S3B). The results showed that mycoplasma from these three cell samples were unclassified species, *M. hyorhinis*, and mixed mycoplasma (unclassified species, *M. hyorhinis*, and *Mycoplasma yeatsii*), respectively (Figure 3A). As seen in Figure 3B, subsequent tests demonstrated that the A15-1 aptamer bound the mixed mycoplasma-infected Jeko-1 cells weakly and did not bind the unclassified mycoplasma-infected Jeko-1 cells. Likewise, the RFU ratios between A15-1 and L7-2-bound unclassified (Unc) or mixed mycoplasma (yea)-infected cells were nearly identical to those of MP^−^ cells (Figure 3C). These results suggested that the A15-1 aptamer cannot be used to detect other species of mycoplasma-infected cells.

### 2.5. Aptamer Cocktail for Detection of Multiple Infection-Causing Mycoplasma Species

**Figure 4:**
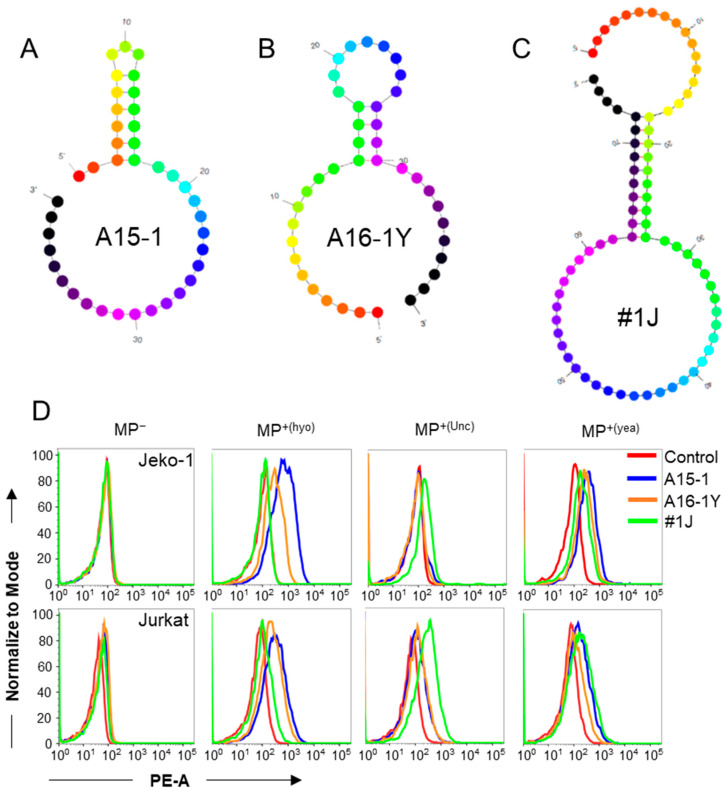
Screening of aptamers for targeting different species of mycoplasma-infected cells. (**A**–**C**) The predicted secondary structures of A15-1 (**A**), newly screened A16-1Y (**B**), and #1J (**C**) aptamers; (**D**) A15-1, A16-1Y, and #1J binding tests on other species of mycoplasma-infected Jeko-1 and Jurkat cells using flow cytometry. The control aptamer is L14-2. MP^−^, mycoplasma negative; MP^+(hyo)^, *M. hyorhinis* positive; MP^+(Unc)^, unclassified mycoplasma negative; MP^+(yea)^, mixed mycoplasma positive. Data show the representative results from three independent experiments.

After determining that A15-1 may be a specific aptamer for *M. hyorhinis* detection, we elected to screen for additional aptamers that may bind other species of mycoplasma-infected cells. We selected two previously synthesized aptamers from our collection [16], A16-1Y and #1J (Table S1), because they had relatively strong binding to mycoplasma-contaminated cells from other laboratories. We predicted the secondary structures of A15-1, A16-1Y, and #1J aptamers (Figure 4A–C). Next, we assessed the binding ability of these three aptamers. We found that both A15-1 and A16-1Y could bind to *M. hyorhinis* and mixed mycoplasma-infected cells, while #1J could bind to unclassified mycoplasma-infected cells, but not *M. hyorhinis*-infected cells (Figure 4D).

**Figure 5:**
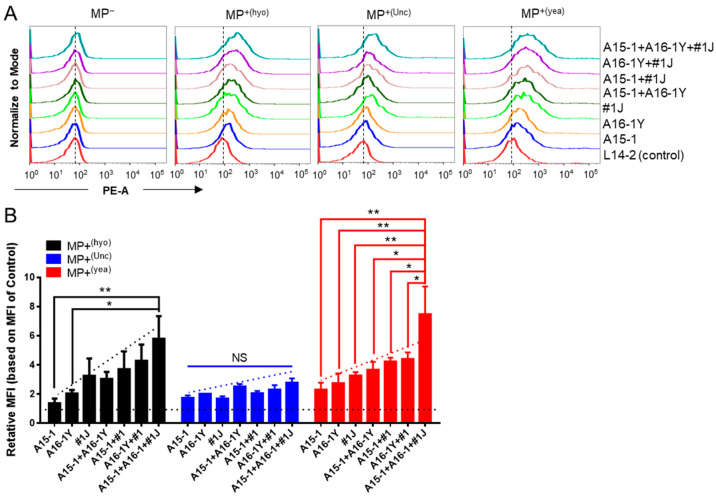
Aptamer cocktail facilitates detection of multiple infection-causing mycoplasma species. The binding ability of the aptamer cocktail is shown for multiple species of mycoplasma-infected cells. (**A**) Binding tests for single, pairwise, and triple mixed aptamers on multiple species of mycoplasma-infected Jurkat cells by flow cytometry. The concentration of each aptamer in pairwise and triple mixed aptamers was 50 nM. The vertical dashed lines indicate the position of the fluorescence peak of L14-2-bound cells. Data show representative results from three independent experiments; (**B**) Relative MFI of various aptamer-bound cells versus control aptamer-bound cells. The control aptamer is L14-2; the horizontal dashed line indicates the baseline of the normalized relative MFI; the diagonal dashed lines indicate the variation tendency; MP^−^, mycoplasma negative. MP^+(hyo)^, *M. hyorhinis* positive; MP^+(Unc)^, unclassified mycoplasma negative; MP^+(yea)^, mixed mycoplasma positive. Data show the mean ± SD of three independent experiments. * *p* < 0.05, ** *p* < 0.01, “NS” means not significant.

We hypothesized that mixed aptamers may detect more species of mycoplasma. We used MP^−^ and MP^+^ Jurkat cells to test the binding ability of pairwise aptamers and triple aptamers. None of the tested aptamers could bind to MP^−^ cells. Pairwise aptamers showed stronger binding to MP^+^ cells than single aptamers but weaker binding than the triple mixed (cocktail) aptamers (Figure 5A). Converting these data to relative mean fluorescence intensity (MFI) confirmed that the aptamer cocktail, comprised of A15-1, A16-1Y, and #1J, possessed the strongest binding ability compared to either pairwise or single aptamers, suggesting that the aptamer cocktail could be used to detect multiple species of mycoplasma (Figure 5B).

**Figure 6:**
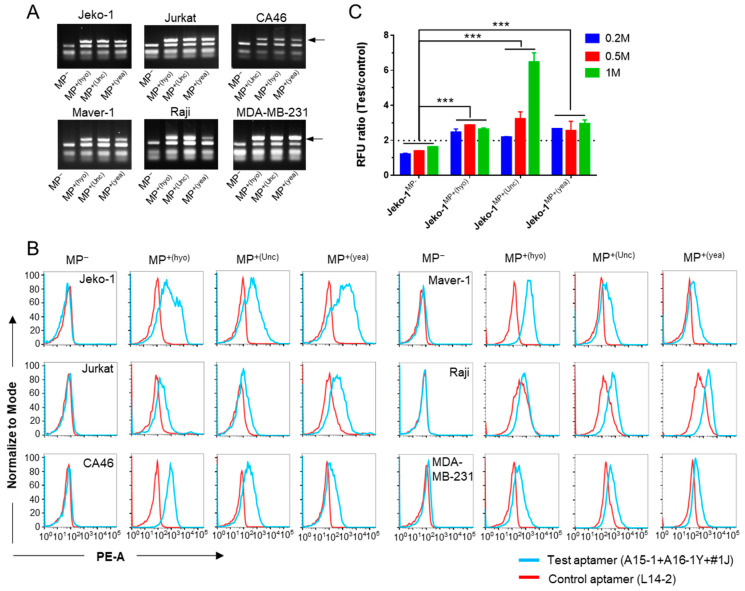
Application of the aptamer cocktail for detection of multiple infection-causing mycoplasma species. (**A**) PCR assays to verify mycoplasma infection status. PCR products were analyzed using 2% agarose gel electrophoresis. Arrows show the PCR amplicons of mycoplasma DNA; (**B**) Histogram plots of flow cytometry results show test (cocktail) or control (L14-2) aptamer binding in MP^−^ or MP^+^ cells. Data show representative results from three independent experiments. (**C**) Microplate reader detection of mycoplasma-infected Jeko-1 cells using the test (cocktail) and control (L7-2) aptamers. Different counts (0.2, 0.5, or 1 million) of Jeko-1 cells were tested. The horizontal dashed line is the cut-off value line that demarcates MP^−^ and MP^+(hyo)^ cells. Data show the mean ± SD of three independent experiments. *** *p* < 0.001. MP^−^, mycoplasma negative; MP^+(hyo)^, *M. hyorhinis* positive; MP^+(Unc)^, unclassified mycoplasma negative; MP^+(yea)^, mixed mycoplasma positive.

We next tested the binding of the aptamer cocktail with various cells. After confirming mycoplasma infection using a commercial PCR assay (Figure 6A), we tested the binding ability of the aptamer cocktail to the infected cells. Compared to the control aptamer, the aptamer cocktail demonstrated stronger binding to MP^+^ cells but equal binding to MP^−^ cells (Figure 6B). Further, we observed binding of the aptamer cocktail to MP^+^ cells under a fluorescence microscope and found that the aptamer cocktail, but not the L14-2 aptamer, could bind to either *M. hyorhinis*, unclassified mycoplasma, or *M. yeatsii* (mixed) mycoplasma-contaminated MDA-MB-231 and Jeko-1 cells, which were observed as red dots (Figure S4). We also detected mycoplasma contamination using a microplate reader. As seen in Figure 6C, the RFU ratio between the test (cocktail) and control aptamer-bound cells was lower than 2 in MP^−^ cells but higher than 2 in MP^+^ cells, suggesting that the cocktail and control aptamers can be used to detect cell contamination from *M. hyorhinis*, unclassified mycoplasma, and *M. yeatsii* (mixed).

### 2.6. Competitive Binding of A15-1, A16-1Y, and #1J Aptamers to Mycoplasma-Infected Cells

**Figure 7:**
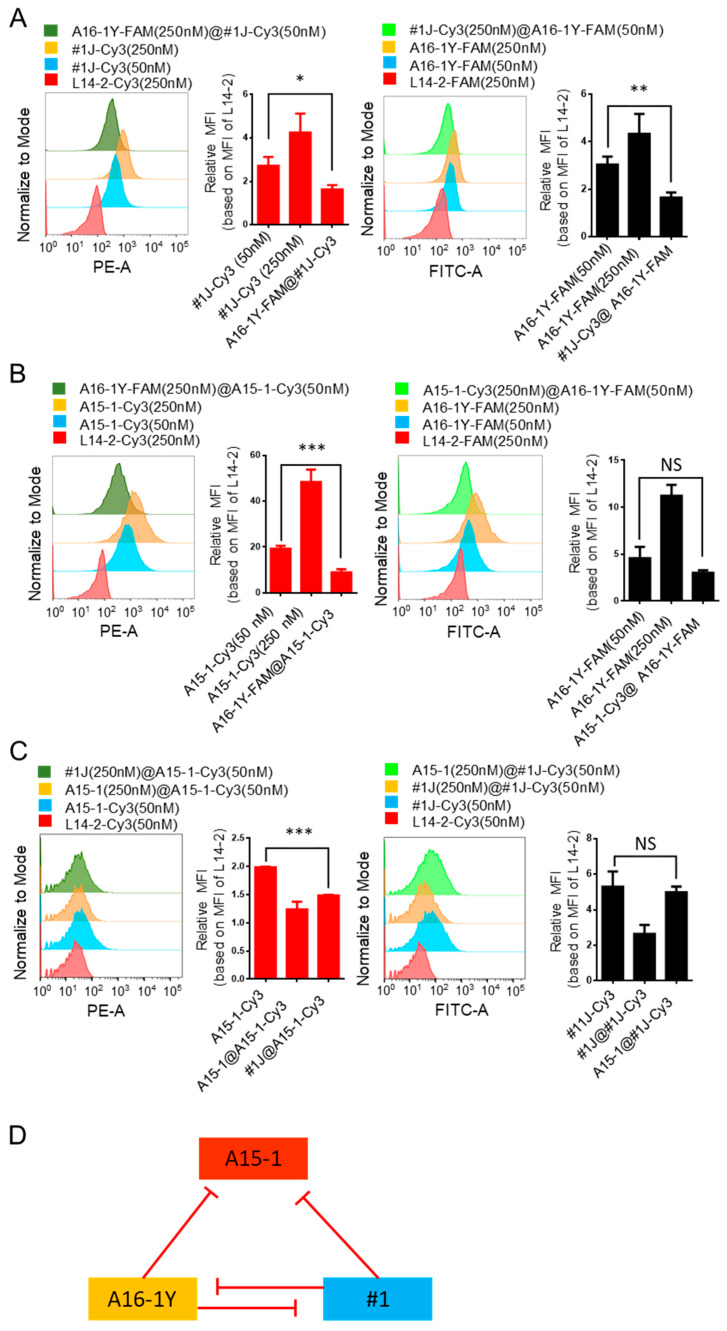
Competitive binding of A15-1, A16-1Y, and #1J aptamers to mycoplasma-infected cells. Mixed mycoplasma-infected Jeko-1 cells were pre-incubated with [250 nM] blocking aptamer for 10 min, then incubated with 50 nM testing aptamer for 15 min. Then, MFI of the test aptamer-bound cells were calculated and compared. Histogram plots of flow cytometry results are presented on the left and the corresponding quantifications are presented on the right. (**A**) Competitive binding test between A16-1Y and #1J aptamers; (**B**) competitive binding test between A16-1Y and A15-1 aptamers; (**C**) competitive binding test between A15-1 and #1J aptamers. (**D**) Diagram of the competitive binding between A15-1, A16-1Y, and #1J aptamers. Red lines indicate inhibition. “@” in the figure legends indicates the separator between blocking and testing aptamer; the aptamers before “@” are blocking aptamers and those after “@” are testing aptamers. Data show the mean ± SD of three independent experiments. * *p* < 0.05, ** *p* < 0.01, *** *p* < 0.001, “NS” means not significant.

Binding between aptamers and their targets is determined by their three-dimensional interactions, including hydrogen bonding, shape compatibility, base stacking, electrostatic and hydrophobic interactions, and van der Waals forces [14,18]. Although we found the binding ability of the aptamer cocktail to be stronger than that of the single aptamers, we further sought to determine whether there were competitive bindings among the three aptamers. Therefore, we conducted competitive binding tests using mixed mycoplasma-infected Jeko-1 cells. As seen in Figure 7A, the binding ability of #1J was significantly reduced by A16-1Y blocking. Similarly, the binding ability of #1J was significantly reduced by A16-1Y blocking. As seen in Figure 7B,C, the binding ability of A15-1 was significantly reduced by A16-1Y or #1J blocking, but the binding ability of A16-1Y or #1J was not affected by A15-1 blocking. Thus, A16-1Y and #1J inhibit each other on the targeted binding, A15-1 binding can be reduced by either A16-1Y or #1J, and A15-1 does not inhibit A16-1Y or #1J (Figure 7D).

### 2.7. Detection of Mycoplasma-Contaminated Culture Medium

**Figure 8:**
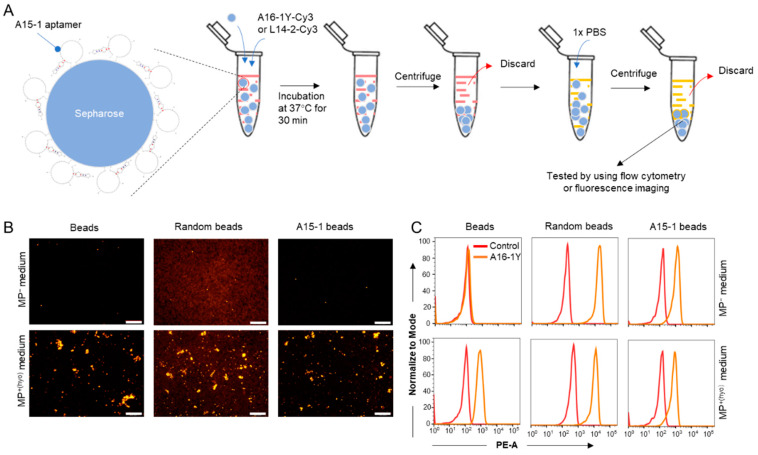
Detection of mycoplasma-contaminated culture medium. Streptavidin-immobilized sepharose and the A16-1Y aptamer were used to distinguish between MP^+^ and MP^−^ cell culture media. (**A**) Flow chart of the experiments for mycoplasma detection in cell culture medium. Indicator beads were incubated with MP^−^ or MP^+^ cell culture medium containing [100 nM] Cy3-labeled A16-1Y or control aptamer at 37 °C for 30 min. Then, the fluorescence of those aptamer-bound beads was observed using a fluorescent microscope or a flow cytometer. Red in the tube indicates cell culture medium, yellow in the tube indicates 1 × PBS. (**B**) Fluorescence microscopy observation of the Cy3-labeled A16-1Y aptamer binding with *M. hyorhinis*-adherent sepharose. (**C**) Histogram plots of flow cytometric data representing Cy3-labeled A16-1Y aptamer binding with *M. hyorhinis*-adherent sepharose. Scale bar = 500 μm.

Because A15-1 did not inhibit the binding of either A16-1Y or #1J, we hypothesized that A15-1-linked beads could bind and capture *M. hyorhinis* from *M. hyorhinis*–contaminated liquid samples (e.g., cell culture medium and serum). To test this, we synthesized biotin-labeled A15-1 and then linked it to streptavidin-immobilized sepharose (A15-1 beads). As a negative control, we synthesized a biotin-labeled N_40_ ssDNA library oligo and linked it to streptavidin-immobilized sepharose (random beads). The original streptavidin-immobilized sepharose (beads) served as a vehicle control. First, we tested whether A15-1 beads were able to gather mycoplasma specifically, following the procedures illustrated in Figure 8A. We found that A15-1 beads could not enrich mycoplasma compared to beads or random beads (Figure S5A). Interestingly, after subjecting beads to three additional washes, we found they showed a stronger attraction to mycoplasma than the random or A15-1 beads, indicating that mycoplasma can adhere to streptavidin-immobilized sepharose directly (Figure S5B). Furthermore, the sepharose-adherent mycoplasma was observed and confirmed using scanning electron microscopy (SEM) (Figure S5D).

Next, we determined whether the sepharose-adherent mycoplasma could be detected by A15-1, A16-1Y, or #1J aptamers. Specifically, we questioned whether A15-1, A16-1Y, or #1J aptamers could bind to streptavidin-immobilized sepharose. Our results revealed that #1J, but not A15-1 or A16-1Y aptamers, can bind to fresh streptavidin-immobilized sepharose (Figure S5E). As such, either A15-1 or A16-1Y could be used as a detection aptamer. Fluorescence imaging and flow cytometry results indicated that random beads and A15-1 beads could nonspecifically bind to the A16-1Y aptamer (Figure 8B,C and Figure S5F), suggesting that neither random beads nor A15-1 beads can be used to detect mycoplasma in cell culture medium. However, streptavidin-immobilized sepharose was able to capture and detect planktonic mycoplasma in cell culture medium. We used streptavidin-immobilized sepharose and the A16-1Y aptamer to detect mycoplasma contamination in cell culture medium of suspended (Jeko-1 and Jurkat cells) and adherent cells (MCF-7 cells) (Figure S5C,G). We also tested whether streptavidin-immobilized sepharose and the A16-1Y aptamer could distinguish between mycoplasma-positive (MP^+(hyo)^) and mycoplasma negative (MP^−^) cell culture medium but determined that the method was unstable for mycoplasma detection in liquid samples.

## 3. Discussion

Mycoplasma contamination is increasingly recognized as a harmful and invisible threat to cell cultures. This recognition has led to the emergence of a multitude of mycoplasma detection methods. Most make use of DNA amplification techniques that are characterized as stable, specific, and rapid, such as general and real-time PCR assays [6,19,20,21], Cycleave PCR [22], and loop-mediated isothermal amplification [23]. Some are based on immunological principles, such as enzyme-linked immunosorbent assays [24,25], and some are based on microscopic techniques, including electron microscopy and fluorescence microscopy [26,27,28]. Other detection methods include Fourier transform infrared microspectroscopy [29], biochemical tests [30], and *Gaussia* luciferase-based assays [31]. All aim to establish a rapid, simple, and inexpensive mycoplasma detection scheme. Here, we describe a feasible mycoplasma detection technique using ssDNA aptamer probes that is based on previous findings from our laboratory [16].

Aptamers have been broadly applied to pathogen detection [10,32] and cancer diagnostic endeavors [33,34] in the past. Mycoplasma contamination differs from other bacterial contaminations in several ways. Mycoplasma lack a cell wall (and thus, are not affected by traditional antibiotics), have limited biosynthetic capabilities (and thus, act as parasites on their host), and are mainly located extracellularly. This last difference is significant, as it indicates that mycoplasma-contaminated cells can be bound by mycoplasma-specific aptamers that can then be detected by flow cytometry. We have successfully reduced both the time and economic cost of this method by optimizing the binding buffer, reaction time, and concentration of the aptamer probes. The binding buffer used in previous binding tests was RPMI-1640 cell culture medium containing 1 mg/mL BSA and 0.1 mg/mL tRNA. In the present study, we determined that the A15-1 aptamer binds with *M. hyorhinis*-infected cells more efficiently in a 1 × PBS solution lacking Mg^2+^ and Ca^2+^. This solution is more economical than the previous binding buffer. Magnesium ions, though typically critical for the structure formation of RNA and DNA aptamers [35,36,37], did not affect the binding ability of A15-1 aptamer, suggesting that the structure formation of A15-1 is less affected by magnesium ion. We further found that tRNA, but not BSA, affected the binding of the A15-1 aptamer to its target (Figure S1), suggesting that tRNA may nonspecifically impede A15-1 binding ability. We found that binding time did not affect the binding ability of A15-1 significantly, suggesting that 10 min is enough for A15-1 aptamer binding.

We confirmed that the A15-1 aptamer binds MP^+(hyo)^ but not MP^−^ cells but wanted to determine how to estimate whether a suspected cell sample is MP^+^ or MP^−^ using the A15-1 aptamer. Because cells may nonspecifically absorb nucleotide acids [13], A15-1 may nonspecifically adhere to some cells, leading to a pseudo-positive in mycoplasma detection using only the A15-1 aptamer probe. Therefore, it is important to select a control aptamer that does not bind (or only weakly binds) with cells to control for nonspecific cell binding. We used the L14-2 aptamer, which was selected from a library of previously synthesized aptamers in our laboratory. For flow cytometry experiments, Cy3-labeled L14-2 and A15-1 aptamers work well to distinguish MP^+(hyo)^ from MP^−^ cells, according to their different binding abilities to MP^+^ cells.

To simplify the detection procedure, we investigated whether we could detect mycoplasma contamination using a fluorescence microplate reader. By using the RFU ratio between A15-1 and L14-2 bound cells, we found that there was no cut-off value with which MP^+(hyo)^ and MP^−^ cells could be differentiated; this was because the fluorescence intensity of A15-1- but not L14-2-bound cells increased with increasing cell counts. Therefore, we determined that the ideal control aptamer should weakly bind to MP^+^ cells and not bind to MP^−^ cells. On this principle, we selected L7-2 as the control aptamer for microplate reader experiments. Together, A15-1 and L7-2 aptamers allowed us to distinguish between MP^+(hyo)^ and MP^−^ cells. Of note, 0.01% Tween-20 should be supplied in the wash buffer to remove nonspecifically attached aptamers, which can significantly affect results [38]. Relative to traditional mycoplasma PCR detection assays, the detection method presented herein provides results more rapidly (30 min), and at less expense (less than $0.5 USD/test).

Because aptamer-target binding is highly specific, we considered that A15-1 may not bind to cells contaminated by other species of mycoplasma. We confirmed this suspicion using cells contaminated by mycoplasma from other laboratories (Figure 3) and concluded that the A15-1 aptamer likely binds with a specific component of *M. hyorhinis*. To detect a broader range of mycoplasma species, we sought to develop other testing aptamers. We selected A16-1Y and #1J aptamers from our aptamer library. After we mixed these two aptamers with A15-1, we found that the aptamer cocktail could not only bind and distinguish three different species of mycoplasma in contaminated cells but also that it had enhanced binding ability for MP^+^ cells (Figure 5). Our experimental design was similar to that of a previous study, wherein use of an aptamer cocktail was identified as a method for enhancing aptamer sensitivity [39] and enhanced the detection signal of bacteria [40]. Further, we demonstrated that the aptamer cocktail described herein can be used to distinguish between MP^+^ and MP^−^ cells using a microplate reader, a flow cytometer, or a fluorescence microscope.

Some aptamers were applied to detect microorganisms in liquid samples using a double-aptamer sandwich enzyme-linked oligonucleotide assay (ELONA) [10]. We tested whether these three aptamers could be applied in ELONA to detect mycoplasma in liquid samples. We found that A16-1Y and #1J mutually inhibited the binding ability of the other, but that the A15-1 aptamer did not inhibit the binding ability of A16-1Y or #1J aptamers. To investigate whether the competitive binding could be ascribed to similar motifs among the three aptamers, we performed a sequence alignment analysis and found that there were three similar motifs between A16-1Y and #1J, namely, “GNGTGNNGNNG”, “TNNCTA”, and “ANGGNGNCNTNGT”; two similar motifs between A16-1Y and A15-1, namely, “GTGGGGTTG” and “GGNNNANGGNGTNNNNGTG”; two similar motifs between A15-1 and #1J, namely, “GTGNNGNNG” and “ACNCNGGANANGGTGNNTNNGT”; and two similar motifs among A15-1, A16-1Y, and #1J, namely, “GTGNNGNNG” and “GNNNNNGTG” (Figure S6). The results imply that one of the necessary motifs for MP+ cell binding is “GTGNNGNNG,” which exists in the three aptamers. Another is a combination motif containing “TNNCTA” and “ANGGNGNCNTNGT,” which exists in A16-1Y and #1J aptamers but not in the A15-1 aptamer. These two essential motifs may cause the competitive relationship observed among the A15-1, A16-1Y, and #1J aptamers.

Because the A15-1 aptamer did not inhibit the binding ability of the A16-1Y or #1J aptamer, we hypothesized that a biotin-conjugated A15-1 aptamer linked to solid streptavidin-immobilized sepharose could capture planktonic mycoplasma, allowing mycoplasma-adherent sepharose to be detected. Using a PCR detection assay and SEM observation, we found that the sepharose could nonspecifically absorb mycoplasma and that the linked A15-1 did not increase the adhesion efficiency. We modified the 5′-terminal of the A15-1 aptamer with biotin and there was no space-linker between the biotin and the aptamer. When the A15-1 aptamers were immobilized onto the sepharose, their orientations were immobilized and even their structures may be changed as well. In contrast to free A15-1 aptamers, without a space linker, the binding-functional regions of immobilized A15-1 aptamers may be sterically hindered, which leads to the loss of binding ability of A15-1 aptamers [41]. This design should be further optimized to determine whether the immobilized A15-1 aptamer can capture planktonic mycoplasma.

Another issue is that the A16-1Y aptamer can bind to the A15-1-immobilized sepharose. Considering these drawbacks, we next used streptavidin-immobilized sepharose to capture planktonic mycoplasma and detected mycoplasma using the A16-1Y aptamer. Our results showed that mycoplasma could be detected using this method. However, we found this method unstable for mycoplasma detection in cell culture supernatant, due to difficulties with obtaining reproducible results. There are two possible reasons for this difficulty. One involves the inherent randomness and low efficiency of mycoplasma adhesion to streptavidin-immobilized sepharose, which results in an unknown amount of adherent mycoplasma and variable adhesion properties. In other words, because the exact number of mycoplasma in the liquid sample is unknown, samples with higher quantities of mycoplasma may attach to cells to facilitate successful detection, while those with lower quantities do not. Another involves nonoptimal binding conditions for the A16-1Y aptamer. We added A16-1Y aptamer into cell culture supernatant directly, but FBS, ionic strength, pH, or other factors may affect the binding affinity of this aptamer to target cells [42]. Further, planktonic mycoplasma may outcompete the A16-1Y aptamer for binding sites, resulting in less A16-1Y aptamer binding with sepharose-adherent mycoplasmas. With refinement, we believe the detection model presented herein should be feasible, particularly in light of the success of similar approaches using magnetic beads coated with aptamers designed to capture *Trypanosoma cruzi* in the blood [43]. We suggest that solid particles and aptamers with a stronger binding ability to various mycoplasmas should be further explored and selected in the future.

In summary, we have used ssDNA aptamers to develop novel mycoplasma detection methods compatible with flow cytometers, fluorescence microscopes, or microplate readers. Although the current application can be improved upon, it is of great value to be further developed. Application of the detection system and model presented herein will facilitate mycoplasma detection using aptamer technology that is simpler, cheaper, and faster than existing methods.

## 4. Materials and Methods

### 4.1. Cell Culture

The following cell lines were used: Burkitt’s lymphoma cell lines CA46 and Raji; Mantle cell lymphoma cell lines Jeko-1, Maver-1, and Mino; Hodgkin lymphoma cell lines HDLM-2 and KM-H2; T leukemia cell line Jurkat; and breast cancer cell lines MDA-MB-231 and MCF-7. HDLM-2 and KM-H2 cell lines were kind gifts from Dr. Barbara Savoldo at Baylor College of Medicine (Houston, TX, USA). All other cell lines were purchased from American Type Culture Collection (ATCC, Manassas, VA, USA). All lymphoma and leukemia cells were cultured in RPMI-1640 cell culture medium (Corning, Manassas, VA, USA) containing 10% fetal bovine serum (FBS; Corning, Manassas, VA, USA) and 100 IU/mL penicillin-streptomycin (GE Healthcare, Pittsburgh, PA, USA). MDA-MB-231 and MCF-7 cells were cultured in DMEM cell culture (Corning, Manassas, VA, USA) containing 10% FBS and 100 IU/mL penicillin-streptomycin. Cells were incubated at 37 °C with 5% CO_2_ and 95% humidity in a CO_2_ incubator (MCO-19AIC(UV), SANYO, Osaka, Osaka, Japan). Trypsin (0.25%) (Corning, Manassas, VA, USA) was used to dissociate MDA-MB-231 and MCF-7 cells for passage.

### 4.2. Mycoplasma Infection

MP^+^ cells were harvested and centrifuged at 500× *g* for 10 min. The supernatant was collected and hermetically stored at 37 °C as the source of mycoplasma contamination. For artificial mycoplasma contamination, healthy cell lines were cultured in a 25 cm^2^ flask with 7 mL of appropriate culture medium and then infected with 0.2 mL of the stored mycoplasma-containing supernatant. The infected cells were then cultured for three days without changing the medium. Meanwhile, mycoplasma negative cells were cultured in another CO_2_ incubator with the same culture condition. After that, the infection status was checked using e-Myco mycoplasma PCR detection kits (v2.0) (iNtRON Biotechnology, Kirkland, WA, USA). The MP^+^ cells were kept for continued cultivation.

### 4.3. Mycoplasma Detection by PCR Assay

All detection procedures were conducted according to the manual of the e-Myco mycoplasma PCR detection kit. Briefly, more than 5000 cells (or 10 μL of cell culture supernatant) were collected. Cells were washed with 1 × PBS once and then resuspended with 100 μL of 1 × PBS (Corning, Manassas, VA, USA). The cell culture supernatant samples or resuspended cell samples were then boiled at 95 °C for 5–10 min and centrifuged at 12,000 rpm for 2 min. The supernatant was used as template for PCR assays. PCR cycling parameters were set as follows: 95 °C for 1 min; 35 cycles of 95 °C for 30 s and 60 °C for 20 s, and then 72 °C for 30 s; 72 °C for 2 min for the final extension. PCR products were analyzed by using 2% agarose gel electrophoresis.

### 4.4. Mycoplasma Species Identification

MP^+^ Jeko-1 cells were harvested for whole DNA isolation using an Allprep DNA/RNA Mini kit (Qiagen, Hilden, Germany), according to the kit manual. The quality of the isolated DNA was tested by using agarose gel electrophoresis and a spectrophotometer (Nanodrop 2000, Thermo Fisher Scientific, Waltham, MA, USA). DNA samples were sent to GENEWIZ (South Plainfield, NJ, USA) for the 16S-EZ NGS analysis.

### 4.5. Flow Cytometry Analysis

To test the binding of aptamers with MP^+^ or MP^−^ cells, flow cytometry was performed. Cy3-labeled aptamers (synthesized by Integrated DNA Technologies, Coralville, IA, USA) were diluted in binding buffer and heated at 95 °C in a dry bath for 5–10 min. The aptamer-containing binding buffer was taken out to cool at room temperature. Then, 100 μL of different concentrations of aptamers were incubated with cells at room temperature. Cells were washed twice with wash buffer and resuspended in 1 × PBS containing 2% FBS for flow cytometry analysis with a FACScan cytometer (FACS Fortessa, BD Biosciences, San Jose, CA, USA). Data were analyzed using FlowJo v10.0.7 software (FlowJo, Ashland, OR, USA).

### 4.6. Mycoplasma Detection Using Fluorimetry

MP^+^ or MP^−^ cells were harvested in select counts (0.2, 0.5, 1, 2, or 4 million) following centrifugation at 400× *g* for 2 min. Cells were washed once with 1 × PBS and incubated with 50 nM of pre-heated test aptamer (A15-1, A16-1Y, #1J, or their mixture) or control aptamer in a total volume of 100 μL of 1 × PBS at room temperature for 15 min. After incubation, cells were washed with 1 mL of 0.01% Tween-20 containing 1 × PBS and centrifuged at 400× *g* for 2 min. The supernatant was discarded, and cells were resuspended in 100 μL of 1 × PBS and transferred into the wells of a 96-well black opaque plate. The fluorescence intensities (*F*) of cells were measured by multi-mode microplate readers (Spectramax M2e, Molecular Device, San Jose, CA, USA and Synergy^TM^ H4, BioTek, Winooski, VT, USA) at excitation and emission wavelengths of 540 nm and 575 nm, respectively. The RFU ratio was calculated using the following equation:

(1) $$RFU\;ratio=\frac{F\left(test\right)-F\left(blank\right)}{F\left(control\right)-F\left(blank\right)}$$

### 4.7. Competitive Binding Tests

Competitive binding tests were performed to explore whether different aptamers bind the same molecular target. The competitive binding test between A15-1 and A16-1Y is described here as an example. *M. yeatsii* (mixed)–positive Jeko-1 cells were harvested and then incubated with 250 nM of pre-heated FAM-labeled A16-1J aptamer at room temperature for 10 min, followed by addition of pre-heated Cy3-labeled A15-1 aptamer to a final concentration of 50 nM with continued incubation for 15 min. After incubation, cells were centrifuged at 300× *g* for 2 min and washed once with 1 × PBS. Cells were resuspended in 1 × PBS containing 2% FBS for flow cytometry analysis.

### 4.8. Mycoplasma Detection in Cell Culture Medium Samples

Streptavidin sepharose (100 μL) (GE Healthcare, Chicago, IL, USA) was washed with 1 × PBS twice and resuspended in 1 × PBS to a total volume of 200 μL. *M. hyorhinis*–contaminated and healthy cell culture supernatants were collected after removing cells by centrifugation at 300× *g* for 2 min. Each 250 μL aliquot of collected cell culture supernatant was mixed with 20 μL of prepared streptavidin sepharose and 100 nM of pre-heated Cy3-labeled aptamers and incubated on a gently rocking mixer at 37 °C for 30 min. After incubation, sepharose was washed three times with 1 × PBS. Mycoplasma on the sepharose was detected using PCR assays. Fluorescence intensities of aptamers on the sepharose were measured using the flow cytometer. For microscope observation, the sepharose beads were suspended in 200 μL of 1 × PBS and 100 μL of suspended beads were transferred into different wells of a black, clear bottom 96-well plate (Corning, Manassas, VA, USA). Then, beads were observed and photographed with an Olympus IX81 inverted fluorescence automated live cell microscope (Olympus Corporation, Shinjuku City, Tokyo, Japan) and the cellSens Standard imaging software (version 1.12) (Olympus Corporation, Shinjuku City, Tokyo, Japan).

### 4.9. Mycoplasma Observation Using Scanning Electron Microscopy (SEM)

The prepared streptavidin-immobilized sepharose beads (20 μL) were incubated with cell culture supernatant (250 μL) of MP^+^ or MP^−^ Jeko-1 cells, as detailed above. After three washes with 1 × PBS, beads were fixed with 2.5% formaldehyde/glutaraldehyde (Electron Microscopy Sciences, Hatfield, PA, USA) at 4 °C for 4 h. Thereafter, the beads were washed once with 1 mL of ultrapure water and then resuspended in 40 μL of ultrapure water. For SEM, a drop of sepharose suspension (5 μL) was spotted onto double-sided carbon conductive tape (Cat# 16084-7, Ted Pella, Redding, CA, USA) to immobilize the sample onto an aluminum SEM specimen mount (Cat# 16111, Ted Pella, Redding, CA, USA). Samples were air-dried. Because iridium is an excellent grain-free coating material, the samples were coated with a thin iridium film of 8 nm by using a magnetron sputtering coater (208HR High Resolution Sputter Coater, Ted Pella, Redding, CA, USA) to enhance the SEM image contrast. The working vacuum pressure in the sputter chamber was 7.5 × 10^−3^ Torr. A plate (57 mm × 0.3 mm) iridium target (Cat #91120, Ted Pella, Redding, CA, USA) was mounted on the top magnetic column of the chamber. The distance from the target to the samples was 6.5 cm. To coat the samples, diluted argon gas was introduced into the chamber and a high voltage of 3 kV between the cathode (iridium target) and the anode (sample holder substrate) was applied. During the sample coating, the metal deposit ratio was 0.2 nm/s. All SEM images were taken at 5–15 kV (accelerating voltage) with electron-beam spot size 3 in the Nova NanoSEM 230 (FEI, Hillsboro, OR, USA). The work distance was 5 mm. All samples were observed in a high vacuum pressure (2 × 10^−6^ Torr) chamber at room temperature.

### 4.10. Data Analysis

The secondary structures of aptamers were predicted using Mfold web service (http://unafold.rna.albany.edu/?q=mfold/DNA-Folding-Form) [44]. Graphpad Prism 6.0 software (GraphPad Software, San Diego, CA, USA) was used for statistical analyses and graphical presentations. Paired Student’s *t*-tests were used in the data analysis, and *p*-values < 0.05 were considered to represent statistically significant differences.

## Supplementary Materials

The following are available online at https://www.mdpi.com/1422-0067/21/11/3784/s1, Figure S1: Optimizing cell binding conditions of A15-1 aptamer, Figure S2: Cell count affects relative fluorescence unit ratios between Cy3-labeled A15-1 and control (L14-2) aptamer-bound cells, Figure S3: Verification of mycoplasma contamination and total DNA isolation, Figure S4: Fluorescence imaging for Cy3-labeled aptamer cocktail- or control (L14-2) aptamer-bound MDA-MB-231 and Jeko-1 cells, Figure S5: Streptavidin-immobilized sepharose capture mycoplasma from contaminated cell culture supernatant, Figure S6. Sequence alignment analysis for A15-1, A16-1Y, and #1J aptamers, Table S1: Sequence information of aptamers.

## Abbreviations

BSA, bovine serum albumin; ELONA, enzyme-linked oligonucleotide assay; FBS, fetal bovine serum; MFI, mean fluorescence intensity; MP, mycoplasma; PCR, polymerase chain reaction; RFU, relative fluorescence unit; SELEX, Systemic evolution of ligands by exponential enrichment; SEM, scanning electron microscopy.
