# Supplementary material for: Aptamer Cocktail to Detect Multiple Species of Mycoplasma in Cell Culture
Source: Int J Mol Sci. 2020 May 27;21(11):3784. doi: 10.3390/ijms21113784 (PMC7312096; doi:10.3390/ijms21113784)
Supplement: Supplementary file 1 [file ijms-21-03784-s001.pdf]

Table S1 Sequence information of aptamers.

Aptamer name	Aptamer sequence (5′-3′)	Length (base)
A15-1	GTG GGG TTG AAA ACG CCG GAG AGG GTG TGT GGG TGG GGT A	40
A16-1Y	TGG GTG GGG TTG TCG CTA GGG GTT TAA GGG GTC GTC GTG A	40
#1J	ATC CAG AGT GAC GCA GCA GCC AAC GTG CTT TCT ACC TTA TTT TCC GTC ACT CTC ACT CTG GAC ACG GTG GCT TAG T	76
L14-2	CCG ATT CTA CGG AGT GAA GGC CCT TTA GGG CCG GTA CCG C	40
L7-2	GTA ACC GGT TGT CTG GGG TTT CGG TGT GGG AGA AAG ATG G	40
Random library	ATC CAG AGT GAC GCA GCA-$[N]_{40}$-TGG ACA CGG TGG CTT AGT	76

Note: $[N]_{40}$ indicates 40 random bases; All the labels including fluorescence dyes and biotin are modified at the 5′-terminal of those sequences.

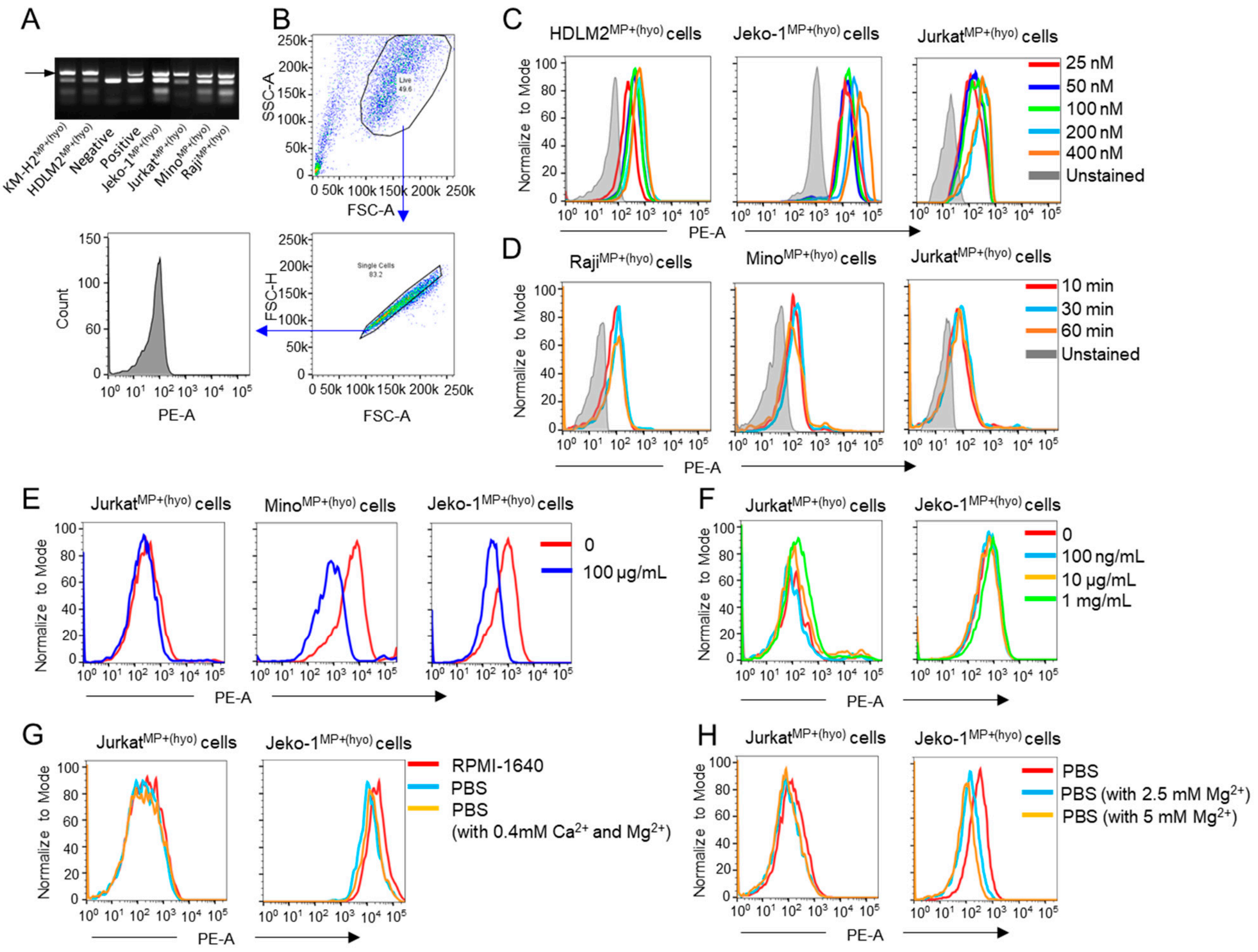

Figure S1. Optimizing cell binding conditions of A15-1 aptamer. The optimal cell binding buffer, bind concentration, and binding time of A15-1 aptamer are 1X PBS (without tRNA, BSA, Mg^{2+}, or Ca^{2+}), 50 nM, and 10 min. (**A**) Mycoplasma-infected cells confirmed by PCR assays. The PCR amplicons were analyzed by 2% agrose gel electrophoresis, and the samples in each lane were indicated below; Arrows shows the PCR amplicons of mycoplasma DNA; (**B**) Gate strategy of flow cytometry assays; (**C**) Test cells were incubated with different concentrations of A15-1 aptamer as indicated in RPMI-1640 binding buffer (RPMI-1640 medium supplied with 1 mg/mL BSA and 0.1 mg/mL tRNA) for 30 min. Resultant cell binding was evaluated by flow cytometry; (**D**) The cells were incubated with 50 nM A15-1 aptamer in RPMI-1640 binding buffer for 10, 30, or 60 min, and changes in cell binding affinity was examined by flow cytometry; (**E-H**) PBS-washed cells were incubated with 50 nM A15-1 aptamer in following binding buffer for 10 min, and then the binding affinity was tested by using flow cytometry: (**E**) RPMI-1640 binding buffer or tRNA-free RPMI-1640 binding buffer; (**F**) RPMI-1640 medium supplied with different concentration of BSA; (**G**) RPMI-1640 medium, PBS, or PBS supplied with 0.4 mM Mg^{2+} and Ca^{2+}; (**H**) PBS, PBS with 2.5 mM or 5 mM Mg^{2+}. Data show the representative results from three independent experiments with similar findings.

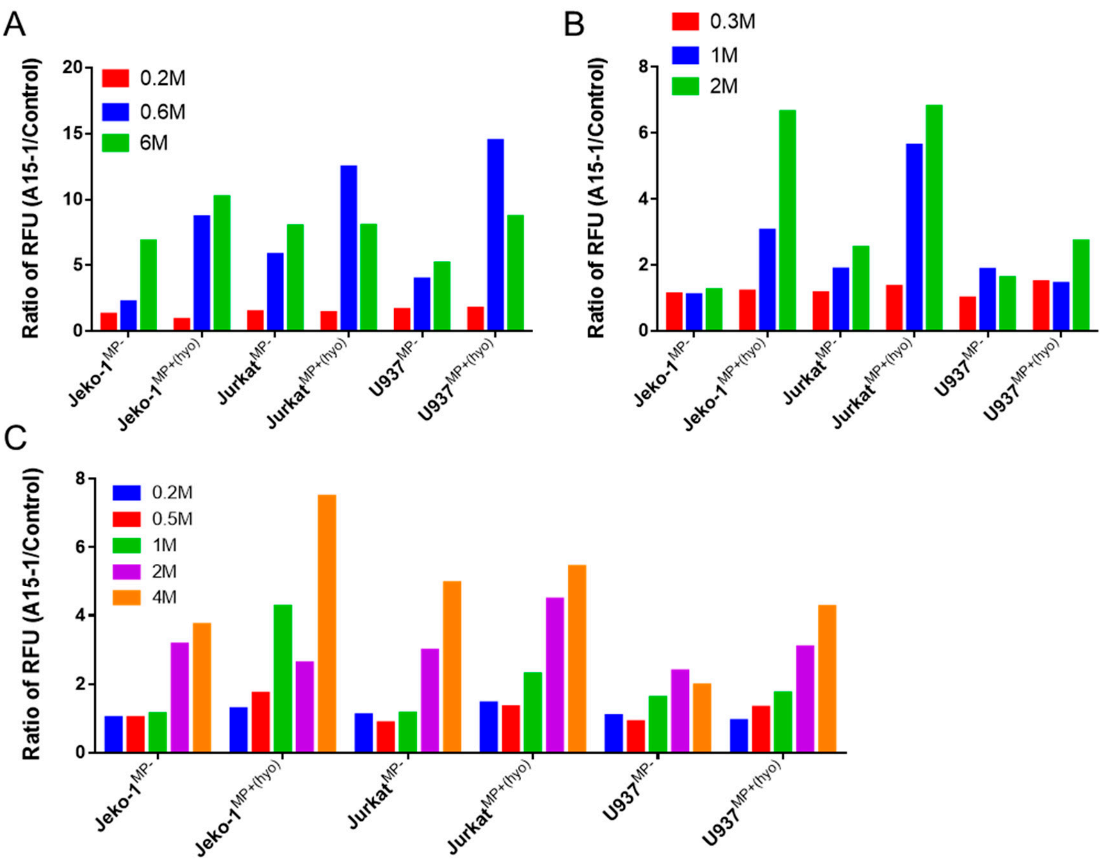

Figure S2. Cell count affects relative fluorescence unit ratios between Cy3-labeled A15-1 and control (L14-2) aptamer-bound cells. Different amounts of *M. hyorhinis* positive ($MP^{+(hyo)}$) or mycoplasma negative (MP^{-}) Jeko-1, Jurkat, or U937 cells were collected and incubated with either Cy3-labeled A15-1 or control aptamer. After washing cells by 0.01% Tween-20 containing 1×PBS once, the fluorescence intensities of those cells were measured using a microplate reader. (**A**) The cell counts are 0.2, 0.6, and 6 million; (**B**) The cell counts are 0.3, 1, and 2 million; (**C**) The cell counts are 0.2, 0.5, 1, 2, 4 million.

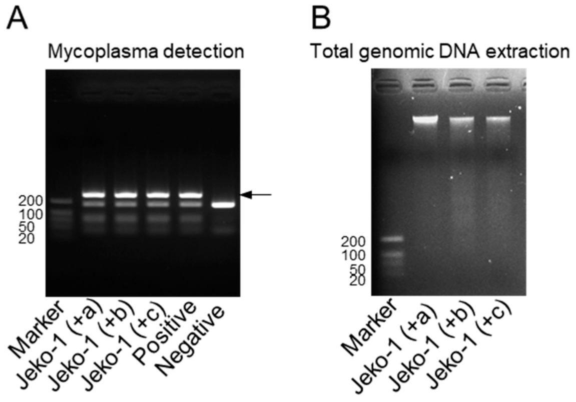

Figure S3. Verification of mycoplasma contamination and total DNA isolation. (**A**) Different species of mycoplasmas were detected using a PCR assay. Arrow shows the PCR amplicons of mycoplasma DNA (**B**) The isolated total DNA including cell DNA and mycoplasma DNA was verified using agarose gel electrophoresis.

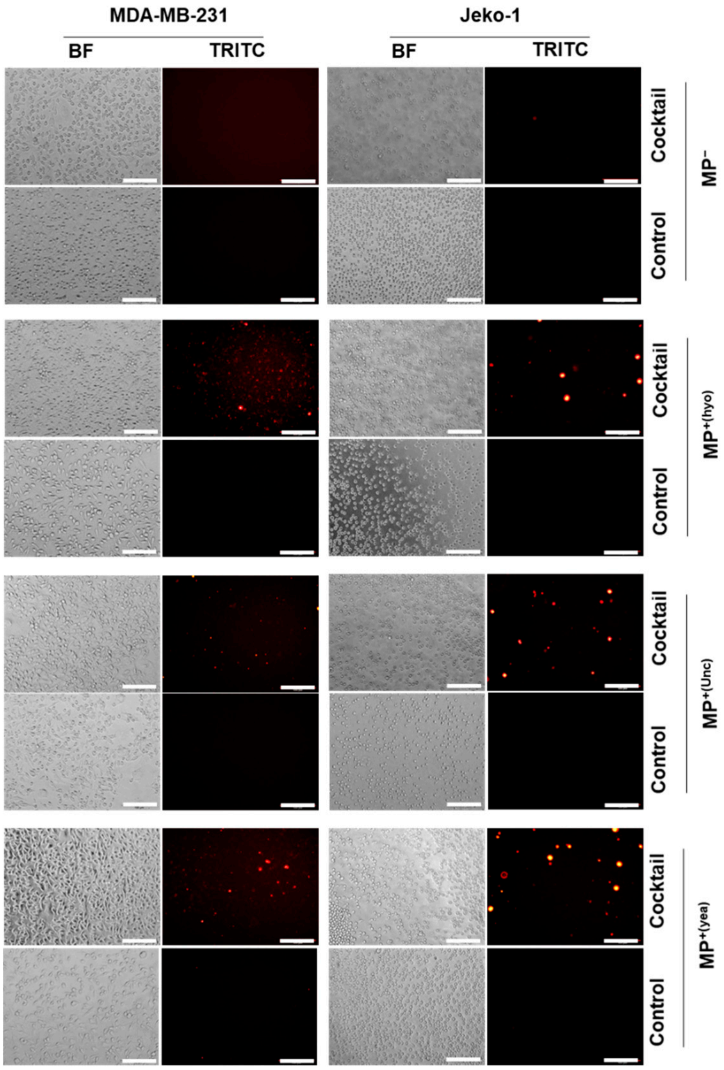

Figure S4. Fluorescence imaging for Cy3-labeled aptamer cocktail- or control (L14-2) aptamer-bound MDA-MB-231 and Jeko-1 cells. MDA-MB-231 cells (1×10^5/well) were seeded into a 48-well plate, and 1×10^6/ml of Jeko-1 cells were seeded into a T25 flask for overnight culture. Then, cells were infected with different species of mycoplasma for further culture. Forty-eight hours later, 2×10^5/sample of Jeko-1 cells were harvested and washed with 1×PBS once, while MDA-MB-231 cells were washed with 1×PBS in wells once. After that, cells were incubated with 100 nM of either Cy3-labeled aptamer cocktail or Cy3-labeled L14-2 in 1×PBS for 10 min, followed by washing with 1×PBS once. Jeko-1 cells were transferred into a 96-well plate. The fluorescence of those cells was observed by using a fluorescence microscope. Scale bar = 100 µm. BF, bright filed; MP^-, mycoplasma negative; $MP^{+(hyo)}$, *M. hyorhinis* positive; $MP^{+(Unc)}$, unclassified mycoplasma negative; $MP^{+(yea)}$, mixed mycoplasma positive

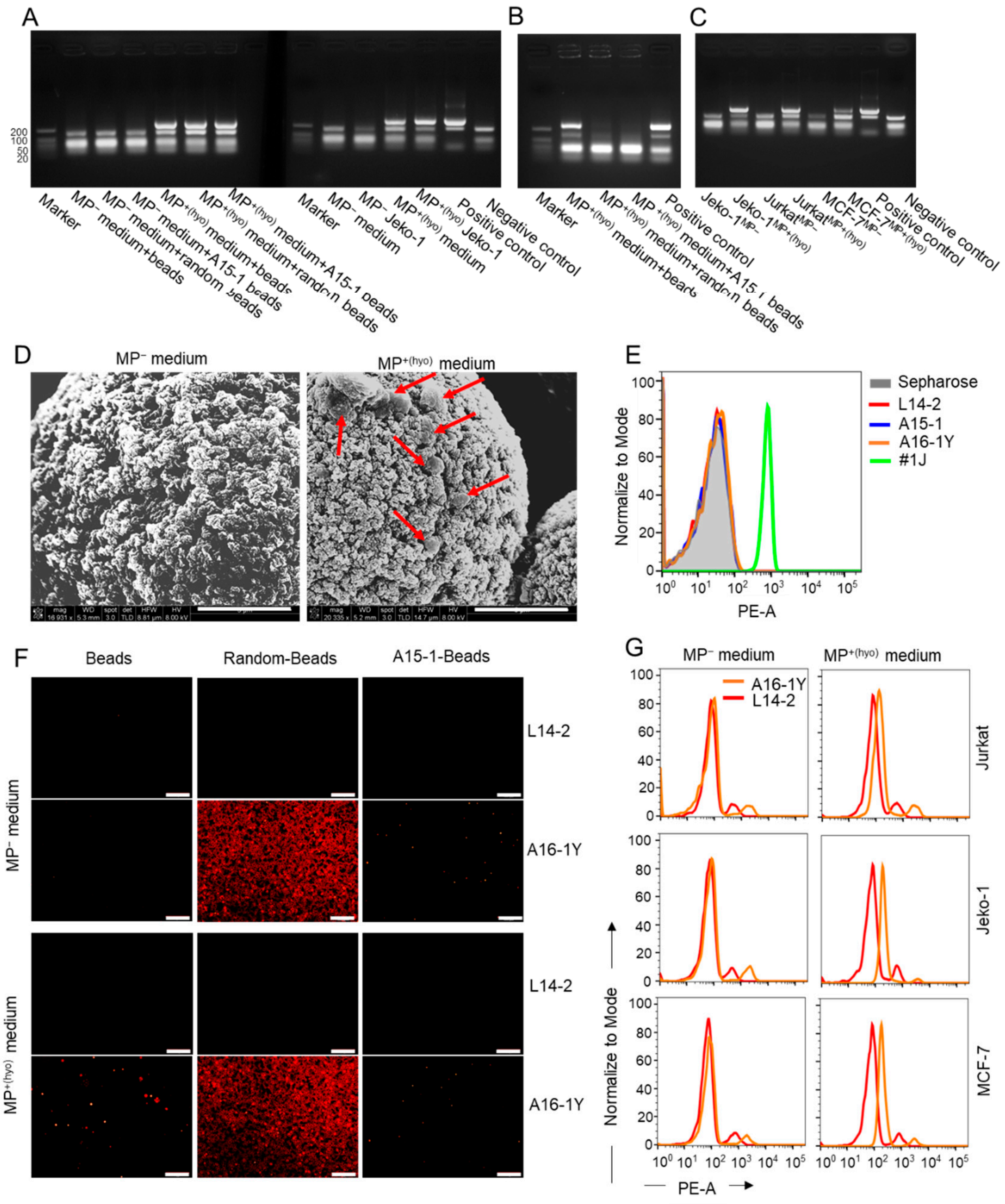

Figure S5. Streptavidin-immobilized sepharose capture mycoplasma from contaminated cell culture supernatant. (**A-C**) Mycoplasma detection for cell culture supernatant and cell culture supernatant-incubated sepharoses using PCR assays; (**D**) SEM micrographs of sepharose-adherent mycoplasma. The scale bar for MP⁻ medium and MP+(hyo) medium is 5 μm. Red arrows show mycoplasma attachment to streptavidin-immobilized sepharose; (**E**) Binding affinity test for L14-2, A15-1, A16-1Y, and #1J aptamers to streptavidin-immobilized sepharose; (**F**) Fluorescence images for *M. hyorhinis*-adherent sepharose after incubating with Cy3-labeled A16-1Y or L14-2 aptamer. Streptavidin-immobilized sepharose (beads), random ssDNA linked sepharose (random-beads), and A15-1 aptamer-linked sepharose (A15-1-beads) were incubated with different cell culture supernatant samples containing either Cy3-labeled A16-1Y or L14-2 aptamer. Fluorescence was observed using a fluorescence microscope. The scale bar for those fluorescence images is 500 μm; (**G**) MP⁻ or MP+(hyo) cell culture medium were incubated with streptavidin-immobilized sepharose and either Cy3-labeled A16-1Y or L14-2 aptamer. The binding of Cy3-labled A16-1Y to the sepharose was tested using a flow cytometer. MP⁻, mycoplasma negative; MP+(hyo), *M. hyorhinis* positive.

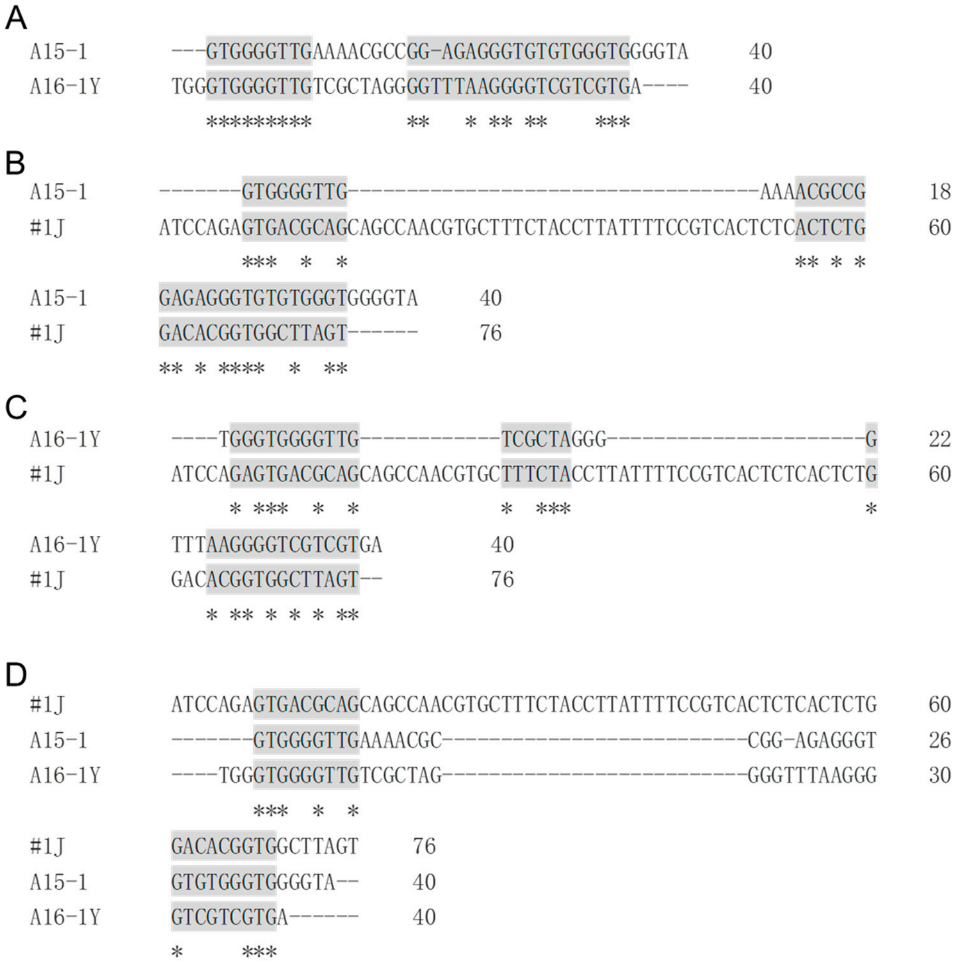

Figure S6. Sequence alignment analysis for A15-1, A16-1Y, and #1J aptamers. Sequence alignment for A15-1 and A16-1Y (**A**), A15-1 and #1J (**B**), A16-1Y and #1J (**C**), and A15-1, A16-1Y and #1J (**D**). Sequences with gray background in each alignment result are the similar motifs. Asterisk indicates the identical bases. Sequences were aligned by using the Clustal Omega (version 1.2.4) online tool (https://www.ebi.ac.uk/Tools/msa/clustalo/).
